# A Nationwide Epidemiological Survey of Adolescent Patients With Diverse Symptoms Similar to Those Following Human Papillomavirus Vaccination: Background Prevalence and Incidence for Considering Vaccine Safety in Japan

**DOI:** 10.2188/jea.JE20210277

**Published:** 2022-01-05

**Authors:** Wakaba Fukushima, Megumi Hara, Yuri Kitamura, Masahiko Shibata, Yoshikazu Ugawa, Koichi Hirata, Akira Oka, Shinya Miyamoto, Susumu Kusunoki, Satoshi Kuwabara, Shuji Hashimoto, Tomotaka Sobue

**Affiliations:** 1 Department of Public Health, Osaka City University Graduate School of Medicine, Osaka, Japan; 2 Research Center for Infectious Disease Sciences, Osaka City University Graduate School of Medicine, Osaka, Japan; 3 Department of Preventive Medicine, Faculty of Medicine, Saga University, Saga, Japan; 4 Division of Environmental Medicine and Population Sciences, Department of Social and Environmental Medicine, Osaka University Graduate School of Medicine, Osaka University, Osaka, Japan; 5 Department of Pain Medicine, Osaka University Graduate School of Medicine, Osaka, Japan; 6 Department of Human Neurophysiology, School of Medicine, Fukushima Medical University, Fukushima, Japan; 7 Department of Neurology, Dokkyo Medical University, Tochigi, Japan; 8 Department of Pediatrics, The University of Tokyo, Tokyo, Japan; 9 Faculty of Human Sciences, University of Tsukuba, Ibaraki, Japan; 10 Department of Neurology, Kindai University Faculty of Medicine, Osaka, Japan; 11 Department of Neurology, Graduate School of Medicine, Chiba University, Chiba, Japan; 12 Department of Hygiene, Fujita Health University School of Medicine, Aichi, Japan

**Keywords:** human papillomavirus vaccine, safety, adverse event, diverse symptoms, adolescents

## Abstract

**Background:**

Since June 2013, Japan has suspended proactive recommendation of human papillomavirus (HPV) vaccination due to self-reported diverse symptoms, including pain and motor dysfunction, as possible serious adverse events following immunization. Although these symptoms may be seen in adolescents without HPV vaccination, their frequency, taking into account disease severity, has not been examined.

**Methods:**

A two-stage, descriptive, nationwide epidemiological survey was conducted in 2016, with a 6-month target period from July 1 to December 31, 2015, to estimate the prevalence and incidence of diverse symptoms among Japanese adolescents without HPV vaccination. Participants were 11,037 medical departments in hospitals selected nationwide by stratified random sampling. Eligible patients had to satisfy four criteria: (1) aged 12–18 years upon visiting hospital; (2) having at least one of four symptoms/disorders (pain or sensory dysfunction, motor dysfunction, autonomic dysfunction, or cognitive impairment); (3) symptoms/disorders persisting for at least 3 months; and (4) both criteria (2) and (3) influence attendance at school or work. We then extracted data of patients with diverse symptoms similar to those after HPV vaccination while considering opinions of doctors in charge.

**Results:**

Estimated 6-month period prevalence of diverse symptoms among girls aged 12–18 years without HPV vaccination was 20.2 per 100,000. Annual incidence was estimated to be 7.3 per 100,000.

**Conclusion:**

Adolescent Japanese girls without HPV vaccination also visited hospitals with diverse symptoms similar to those following HPV vaccination. Our findings predict the medical demands for coincident diverse symptoms, which are temporally associated with but not caused by HPV vaccination of Japanese adolescents.

## INTRODUCTION

Human papillomavirus (HPV) vaccines have been globally available as a primary prevention tool against cervical cancer since their first licensure in 2006. In addition to the well-known efficacy/effectiveness of HPV vaccines in reducing pre-cancerous lesions,^1^ several reports have indicated herd immunity with decreased prevalence of vaccine-targeted HPV genotypes among unvaccinated women.^2^^,^^3^ A recent modelling study suggested that increased HPV vaccination coverage, together with intensive cervical cancer screening, could achieve elimination of cervical cancer by the end of the century.^4^

In Japan, HPV vaccination for girls aged 13–16 years started through a governmental urgent project from November 2010, followed by designation as a national immunization program (NIP) from April 1, 2013, with high vaccine coverage of around 70%. After that, however, “diverse symptoms, including pain and motor dysfunction” (hereafter referred to as “diverse symptoms”), lasting several months and influencing attendance at school or work in some cases, were reported as adverse events following immunization (AEFI). The Ministry of Health, Labour and Welfare (MHLW) of Japan announced suspension of proactive recommendation on June 14, 2013 until they could provide sufficient information about the symptoms. Despite the fact that HPV vaccination is currently still available under NIP, vaccine coverage has severely decreased to around 1%.^5^^–^^8^

Safety of HPV vaccines is well documented by numerous studies that compared vaccinated and unvaccinated individuals and targeted well-known reactions or diseases that were already recognized in medical practice.^1^^,^^9^^–^^13^ However, these reports have not directly answered the claim in Japan that “there have been no diverse symptoms before the introduction of HPV vaccine”.^14^^,^^15^ Alternative studies to address the claim may be newly designed comparative studies with prospective/retrospective information collection on self-reported diverse symptoms. However, unbiased identification of symptoms irrespective of vaccination status would not be achieved because HPV vaccine safety is already a social issue in Japan. Besides, prospective cohort studies are no longer feasible in Japan due to very low vaccination coverage. These considerations cause reluctance to choose comparative study designs for evaluating HPV vaccine safety in Japan.

A lingering key issue is whether or not diverse symptoms can be attributed to diseases that already exist. Although some clinicians mentioned that a certain proportion of adolescents presented diverse symptoms even before the availability of HPV vaccines, few studies have provided quantitative data. A population-based questionnaire survey including 29,846 female residents in Nagoya, Japan, targeted 24 non-specific diverse symptoms rather than specific disease entities and indicated that the cumulative incidence of diverse symptoms among non-vaccinated adolescent girls ranged between 0.2% for loss of ability to walk in a normal way or becoming dependent on a walking stick/wheelchair and 25.6% for menstrual irregularity.^16^ In contrast, according to AEFI data managed by the MHLW Japan, as of November 2014, a total of 2,584 cases (0.08%) were reported among 3,380,000 girls with HPV vaccination, and 186 cases (0.005%) were additionally found to be “not recovered”, with various statuses, including hospitalization, assistant requirement for daily life, or influence on attendance at school or work.^17^ This suggests that the two surveys may have observed different events in terms of disease severity and frequency. In order to reveal the whole picture of more severe (ie, very rare) diverse symptoms that have received social attention in Japan, a nationwide survey is required. Our objective was to conduct a nationwide epidemiological survey to estimate prevalence and incidence of diverse symptoms in Japanese adolescents without history of HPV vaccination, and to obtain background data for considering HPV vaccine safety. Despite not being our original purpose, we also calculated frequency of diverse symptoms among vaccinated adolescents to demonstrate the challenge of comparing frequencies between those with and without HPV vaccination.

## METHODS

### Overview of the survey

This was a descriptive nationwide survey with a two-stage procedure: a first-stage survey estimated the number of patients visiting hospitals, and a second-stage survey revealed patients’ clinical characteristics. We followed “a protocol for a nationwide epidemiological survey on intractable diseases”,^18^^–^^20^ which targeted “rare” diseases.

Sampling frames for participants were 25,325 medical departments in hospitals across Japan, including pediatrics, neurology, anesthesiology (pain clinic), obstetrics/gynecology, orthopedic surgery, gastroenterology, rheumatology, general practice, neurosurgery, and psychiatry/psychosomatic medicine, where adolescents with diverse symptoms were likely to visit. A nationwide hospital database (WELLNESS Co., Ltd., Tokyo, Japan) was used. Additionally, 88 special departments from 83 hospitals that were designated by the MHLW Japan were included in the sampling frames because, as of January 2016, they had to offer clinical management for patients with diverse symptoms after HPV vaccination. From these 25,413 departments, 18,302 (72.0%) were selected by stratified random sampling according to inpatient bed numbers and hospital characteristics (Table 1). Sampling fractions were 100% for departments in hospitals with ≥200 beds, departments in university hospitals, and special departments and 50% for departments in hospitals with <200 beds. We modified the sampling fractions in the original protocol (ie, gradual increase from 5% to 100%) to include many departments because patients with diverse symptoms were expected to be very rare.

**Table 1.** Distribution of departments and number of patients among responded departments with any patients in the first-stage survey

Departments	Number of departments							Reported number of patients	

	Eligible	Sampled (%^a^)	Responded		By reported number of patients				
		
			Number (%^b^)	With any patients	1–9	10–29	≥30^c^	Boys	Girls
Pediatrics	2,596	1,895 (73.0%)	1,294 (68.3%)	131	110	16	5	243	446
Neurology	1,989	1,529 (76.9%)	896 (58.6%)	64	59	4	1	59	164
Anesthesiology (pain clinic)	319	291 (91.2%)	230 (79.0%)	38	36	2	0	18	73
Obstetrics and gynecology	1,861	1,486 (79.8%)	1,037 (69.8%)	12	12	0	0	0	16
Orthopedic surgery	4,780	3,186 (66.7%)	1,949 (61.2%)	39	34	5	0	77	87
Gastroenterology	7,463	4,891 (65.5%)	2,716 (55.5%)	29	26	3	0	40	60
Rheumatology	1,081	774 (71.6%)	469 (60.6%)	18	17	1	0	11	33
General practice	326	293 (89.9%)	168 (57.3%)	11	11	0	0	5	12
Neurosurgery	2,353	1,803 (76.6%)	1,072 (59.5%)	36	35	1	0	53	48
Psychiatry/psychosomatic medicine	2,557	2,066 (80.8%)	1,121 (54.3%)	97	73	17	7	364	552
Special departments in the survey	88	88 (100.0%)	85 (96.6%)	33	28	3	2	33	161
Total	25,413	18,302 (72.0%)	11,037 (60.3%)	508	441	52	15	903	1,652

^a^Number of sampled/Number of eligible.

^b^Number of responded/Number of sampled.

^c^The maximum number was 136 patients.

The first-stage survey started in January 2016. Departments were asked whether patients satisfying criteria visited them during July 1, 2015 to December 31, 2015. Eligible patients had to satisfy four criteria: (1) aged 12–18 years when visiting the departments; (2) having at least one of the following symptoms/disorders: (a) pain or sensory dysfunction, (b) motor dysfunction, (c) autonomic dysfunction, or (d) cognitive impairment; (3) symptoms/disorders persisting for at least 3 months; and (4) both criteria (2) and (3) influence attendance at school or work. These criteria were applied uniformly regardless of the department that was invited for the survey. Survey explanation forms are shown in eMaterials 1. If departments had one or more patients, they were asked to report the number of patients by gender and age (1-year intervals). The numbers of eligible boys and girls were reported. As of 2015, that is, at the time of this survey development, gender neutral HPV vaccination had already been recommended in the United States, Canada, Austria, and Australia.^21^ We thought it would be meaningful to evaluate the background frequency of diverse symptoms for both genders in Japan.

If departments that responded in the first-stage survey had one or more patients that satisfied the criteria, the doctors in charge were invited to the second-stage survey to provide the following clinical characteristics for each patient based on pre-exiting medical charts: previous medical history, date of/age at symptom onset, date of the first/last visit, history of HPV vaccination, presence/absence of each symptom (during July to December 2015), duration of the symptoms, status of schooling/working attendance (during July to December 2015), and diagnoses that were identified/recognized by departments (up to 10 diagnoses, regardless of the cause for the present symptoms, but not providing any diagnoses where examinations were only requested). We also solicited the opinions of doctors in charge about whether they were able to adequately explain the patient’s symptoms during July to December 2015 by their diagnoses (no/yes/unknown), and, if yes, which diagnosis was the most explicable. Informed consent was waived because anonymity was maintained during information collection. The study protocol was approved by the ethics committee at Osaka University, Graduate School of Medicine (No. 15320) and Osaka City University, Graduate School of Medicine (No. 3276).

### Do reported symptoms correspond to “diverse symptoms after HPV vaccination”? Decision process

At the time of information collection, symptoms of the reported patients did not necessarily correspond to those after HPV vaccination. Thus, we further defined whether those symptoms corresponded to “diverse symptoms after HPV vaccination” based on two kinds of information in the second-stage survey: “opinions of doctors in charge”, whether or not they were able to adequately explain patients’ symptoms by the diagnoses that were identified/recognized by the department, and “a diagnosis” that was designated as the most explicable by the doctor in charge (Table 2). Our decision harmonized with a claim in Japan that “there have been no diverse symptoms before the introduction of HPV vaccine”,^14^^,^^15^ and a consensus of the National Expert Committee for Vaccine Safety by the MHLW Japan that diverse symptoms were possibly attributed to a certain mental and physical reaction (referred to as “functional somatic disorder” thereafter^22^). Detailed explanations of the decision process are provided in eMaterials 2. eTable 1, eTable 2, eTable 3, eTable 4, and eTable 5 show the complete list of diagnoses of patients in the second-stage survey.

**Table 2.** Decision process about whether reported symptoms correspond to “diverse symptoms after HPV vaccination”

Answers from doctors in charge in second-stage survey			Decision by the study group whether the reported symptoms correspond to “diverse symptoms”	Frequently reported diagnosis^b^	*n* (%)^c^

Can the doctor adequately explain the patient’s symptoms by the diagnoses?	A diagnosis which is designated as “the most explicable” by the doctor in charge				
Not explicable	—		Correspond	Orthostatic dysregulation, headache, peripheral neuropathic pain	82 (14)

Explicable	The diagnosis *includes* terms such as “due to HPV vaccine” or “after HPV vaccination”^a^		Correspond	Autoimmune encephalopathy (due to HPV vaccine), HPV vaccine- associated neuroimmunopathic syndrome (HANS), HPV vaccine- related neuropathy	31 (5)

	The diagnosis *does not include* terms such as “due to HPV vaccine” or “after HPV vaccination”	Diseases can be clearly distinguished from “diverse symptoms after HPV vaccination”^a^	Don’t Correspond	Systemic lupus erythematosus, epilepsy	21 (4)

		Others^a^	Correspond	Orthostatic dysregulation, adjustment disorder, somatoform disorder	435 (73)

Unknown	—		Unknown	Somatoform disorder, truancy, depression	25 (4)

HPV, human papillomavirus.

^a^Eight clinicians in the study group independently reviewed the list of diagnoses that were designated as “the most explicable” by the doctor in charge for reported patients in the second-stage survey (a total of 201 diagnoses). During the reviews, no other information from the patients was provided. A diagnosis was judged as being clearly distinguished from “diverse symptoms after HPV vaccination” if all eight clinicians judged so.

^b^Complete lists of the reported diagnosis are shown in eTables 1–5.

^c^Denominator of the proportion was 594 patients who were reported to the second-stage survey and whose age at onset was ≥12 years (see eFigure 1 and eTables 1–5).

### Estimation of prevalence and incidence of diverse symptoms

**Figure 1.**
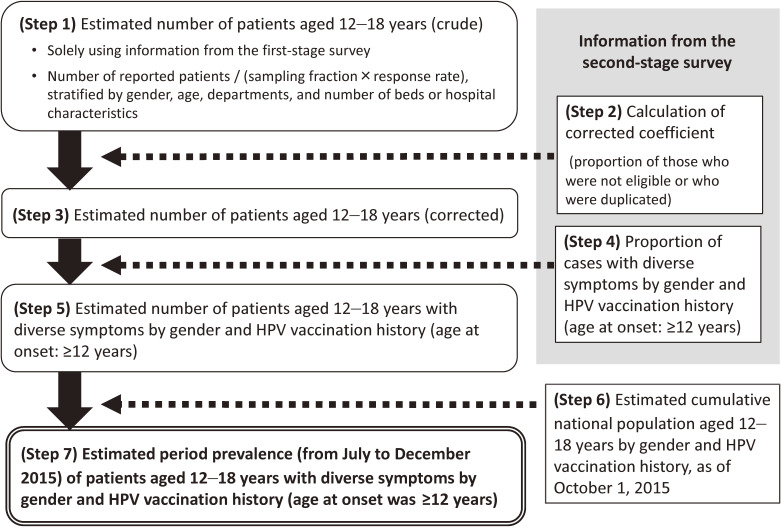
Calculation procedure of estimated period prevalence of diverse symptoms by gender and age. Corrected coefficient was calculated by the number of non-eligible cases (eg, duration of symptoms was <3 months, symptoms did not affect their schooling/working) and duplicated cases (who were identical for gender, date of birth, name initials, prefectures of residence, and where other information was almost the same) as the numerator and the number of reported patients to the second-stage survey as the denominator. HPV, human papillomavirus.

Using information from the first- and second-stage survey, we calculated the period prevalence (from July 2015 to December 2015) of patients aged 12–18 years with diverse symptoms by gender and HPV vaccination history (Figure 1). We limited our estimation to patients whose age at onset was ≥12 years because HPV vaccine is designated as NIP for girls aged 12–16 years in Japan. HPV vaccination history was classified into five categories: “never vaccinated (Group A)”; “vaccinated, symptom occurred before vaccination (Group B)”; “vaccinated, symptom occurred after vaccination (Group C)”; “vaccinated, the time relation between vaccination and symptom onset was unknown (Group D)”; and “unknown vaccination status (Group E)”. All boys were considered “never vaccinated (Group A)”, since HPV vaccine is not designated as NIP for Japanese boys. The data on cumulative number of the vaccinated population aged 12–18 years with at least one dose of HPV vaccine, by gender, was provided by the MHLW. The number of non-vaccinated individuals aged 12–18 years was calculated by subtracting the cumulative number of the vaccinated population from the national population, based on census data (as of October 1, 2015). While we used preliminary national data in reporting our prevalence for the first time at the meeting of National Expert Committee for Vaccine Safety in 2016 and 2017,^23^^,^^24^ finalized national data were used in this report.

We defined “incident patients” as those who experienced onset of diverse symptoms between July and December 2015 (ie, the period identical to the period surveyed). For incidence estimation, we calculated the proportion of incident patients among reported patients in the second-stage survey and multiplied that proportion by the 6-month period prevalence of diverse symptoms.

All calculations were independently performed by two epidemiologists (WF and MH) using SAS software version 9.3 (SAS Institute, Inc., Cary, NC, USA) and cross-checked to guarantee accuracy.

## RESULTS

Of the 18,302 departments selected for the first-stage survey, 11,037 (60.3%) responded (Table 1 and eTable 6). A total of 508 departments reported one or more patients who satisfied inclusion criteria. The reported number of patients was 903 boys and 1,652 girls.

### Estimated number of patients with diverse symptoms and prevalence

The estimated number of patients aged 12–18 years with diverse symptoms, whose age at onset was ≥12 years during the period of July to December 2015, was 829 boys and 1,590 girls (Table 3). The corresponding 6-month period prevalence of diverse symptoms was 19.8 per 100,000 for boys and 40.1 per 100,000 for girls (Table 4). The prevalence for girls was further calculated separately for five categories of vaccination history (Table 5). The estimated number of patients with diverse symptoms and the 6-month period prevalence in Japan were, respectively, 477 and 20.2 per 100,000 for Group A (those never vaccinated), and 445 and 27.8 per 100,000 for Group C (those with symptoms occurring after HPV vaccination). The estimated number of patients with diverse symptoms for Group E (unknown vaccination status) was 604, and prevalence was undetermined because an appropriate denominator was not available.

**Table 3.** Estimated gender- and age-specific number of patients with diverse symptoms during the period of July to December 2015

Age^a^	Reported number of patients in first-stage survey	Estimated number of patients (crude)^b^	Correction coefficient^c^	Estimated number of patients (after correction)				

				All^d^	Patients whose age at onset was ≥12 years^e^			

					Do not correspond to diverse symptoms	Correspond to diverse symptoms	Unknown	Total
Boys								
12 y	99	228	0.69	157	0	54	4	58
13 y	150	345	0.63	217	17	152	0	169
14 y	163	375	0.62	233	8	174	8	191
15 y	150	345	0.58	200	0	171	9	180
16 y	126	290	0.56	162	6	128	6	139
17 y	115	265	0.40	106	9	80	0	89
18 y	100	230	0.46	106	0	84	5	89
12–18 y	903	2,079	0.57	1,185	45	829	36	901

Girls								
12 y	128	295	0.59	174	0	45	0	45
13 y	170	391	0.60	235	0	141	0	141
14 y	231	532	0.68	362	3	268	8	279
15 y	264	608	0.56	340	15	260	18	292
16 y	311	716	0.59	422	11	350	15	376
17 y	315	725	0.58	421	15	334	26	375
18 y	233	536	0.51	273	14	207	14	235
12–18 y	1,652	3,804	0.59	2,244	52	1,590	86	1,728

Note: Estimated number of patients aged 12–18 years do not necessarily correspond to age-specific estimated number of patients due to rounding.

^a^As of October 1, 2015.

^b^Using the information from the first-stage survey, number of reported patients/(sampling fraction × response rate) with stratification by departments and number of beds were calculated within gender- and age-specific strata and summed. See “Step 1” in Figure 1.

^c^Based on information from the second-stage survey. See “Step 2” in Figure 1.

^d^See “Step 3” in Figure 1.

^e^See “Step 4” and “Step 5” in Figure 1.

**Table 4.** Estimated period prevalence (per 100,000 population) of diverse symptoms during the period of July to December 2015, according to gender

Age^a^	Boys			Girls		
	
	Estimated number of patients^b^	Denominator^c^	Estimated period prevalence (per 100,000 population)	Estimated number of patients^b^	Denominator^c^	Estimated period prevalence (per 100,000 population)
12 y	54	567,602	9.5	45	539,923	8.3
13 y	152	584,656	26.0	141	556,169	25.4
14 y	174	593,163	29.3	268	563,639	47.5
15 y	171	607,238	28.2	260	572,575	45.4
16 y	128	607,002	21.1	350	574,169	61.0
17 y	80	615,670	13.0	334	581,725	57.4
18 y	84	605,293	13.9	207	576,615	35.9
12–18 y	829	4,180,624	19.8	1,590	3,964,815	40.1

^a^As of October 1, 2015.

^b^Limited to patients whose age at onset was ≥12 years (after correction, reproduced from Table 3).

^c^The national population in Japan by gender, as of October 1, 2015.

**Table 5.** Estimated period prevalence (per 100,000 population) during the period of July to December 2015 among girls with diverse symptoms, according to HPV vaccination status

Age^a^	All	Group (A)			Group (B) (C) (D)							Group (E)
			
	Estimated number of patients^b^	Estimated number of patients^b^	Denominator^c^	Estimated period prevalence (per 100,000 population)	Estimated number of patients^b^			Denominator^c^	Estimated period prevalence (per 100,000 population)			Estimated number of patients^b^
	
					Group (B)	Group (C)	Group (D)		Group (B)	Group (C)	Group (D)	
12 y	45	30	539,567	5.6	0	0	0	356	0.0	0.0	0.0	15
13 y	141	94	552,476	17.0	0	0	0	3,693	0.0	0.0	0.0	47
14 y	268	126	529,150	23.8	0	21	3	34,489	0.0	60.9	8.7	118
15 y	260	109	326,648	33.4	5	31	8	245,927	2.0	12.6	3.3	107
16 y	350	32	148,695	21.5	4	168	25	425,474	0.9	39.5	5.9	123
17 y	334	30	147,400	20.4	0	150	23	434,325	0.0	34.5	5.3	130
18 y	207	35	119,083	29.4	0	114	0	457,532	0.0	24.9	0.0	60
12–18 y	1,590	477	2,363,019	20.2	16	445	64	1,601,796	1.0	27.8	4.0	604

HPV, human papillomavirus.

Group (A): Never vaccinated.

Group (B): Vaccinated, symptom occurred before vaccination.

Group (C): Vaccinated, symptom occurred after vaccination.

Group (D): Vaccinated, the temporality between vaccination and symptom onset was unknown.

Group (E): Unknown vaccination status.

^a^As of October 1, 2015.

^b^Limited to patients whose age at onset was ≥12 years (after correction, reproduced from Tables 3 and 4).

^c^Estimated cumulative number of national population by gender and HPV vaccination history, as of October 1, 2015.

### Patient characteristics

Of 508 departments that reported one or more patients who satisfied criteria in the first-stage survey, 324 (63.8%) departments responded to the second-stage survey with clinical information on 1,418 patients. The number of patients with diverse symptoms aged 12–18 years whose age at onset was ≥12 years was 183 boys (all were Group A) and 365 girls (Group A: 110, Group B: 2, Group C: 103, Group D: 13, Group E: 137) (eFigure 1). Among girls in Group A, Group C, and Group E, in which the number of patients was not sparse, the proportion having each symptom, the number of symptoms, and the status of schooling/working was evaluated (eFigure 2). Group C showed higher proportions/numbers for most items, whereas Group A and Group E had lower proportions/numbers, but proportions were similar to each other.

### Estimated prevalence of diverse symptoms according to number of symptoms

**Figure 2.**
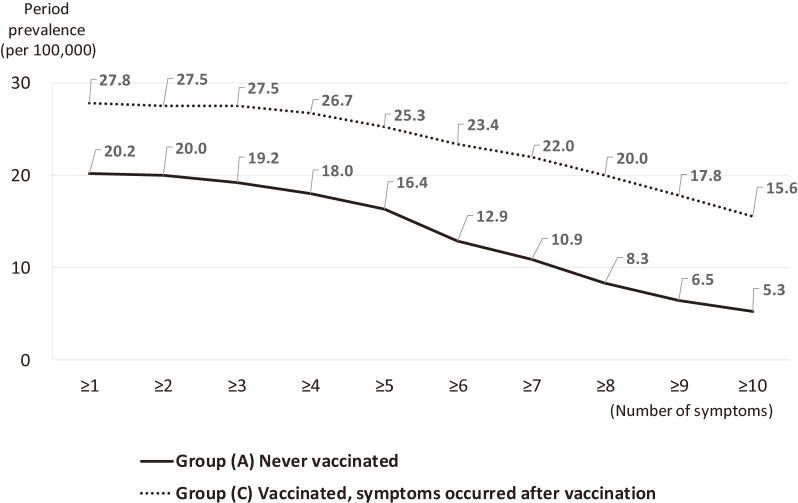
Estimated period prevalence (per 100,000 population) during the period of July to December 2015 among girls with diverse symptoms, according to the number of symptoms.

We calculated period prevalence of diverse symptoms according to the number of symptoms among girls separately for Group A and Group C (Figure 2) using period prevalence (Table 5) and distribution of number of symptoms (eFigure 2, section “v”). Lower prevalence of diverse symptoms was shown among those with a higher number of symptoms. When patients were limited to those whose number of symptoms was ≥10, the 6-month period prevalence among unvaccinated girls (Group A) was 5.3 per 100,000. The most frequent diagnoses among these girls were orthostatic dysregulation for Group A (*n* = 5, reported from five departments, including one patient with postural orthostatic tachycardia syndrome [POTS]), and autoimmune encephalopathy due to HPV vaccine for Group C (*n* = 18, reported from one department).

### Estimated incidence of diverse symptoms among the unvaccinated

Among 110 girls in Group A that were reported to the second-stage survey, 18% (*n* = 20) experienced onset of diverse symptoms between July and December 2015. When we multiplied this proportion (0.18) by the 6-month period prevalence of diverse symptoms in Group A (20.2 per 100,000), the incidence of diverse symptoms among unvaccinated girls was 3.6 per 100,000 during the 6-month period or 7.3 per 100,000 annually. Using the same calculation, the annual incidence of diverse symptoms among unvaccinated girls with ≥10 symptoms was 1.6 per 100,000.

## DISCUSSION

This was the first nationwide survey to estimate prevalence of severe diverse symptoms among adolescents without a history of HPV vaccination in Japan. We confirmed that adolescents with no history of HPV vaccination also visited hospitals due to diverse symptoms and received a wide spectrum of diagnoses, demonstrating that diverse symptoms include elusive and heterogeneous disease entities. We also estimated the annual incidence of diverse symptoms among unvaccinated girls, which predicts the medical demand for diverse symptoms that coincidentally occur following vaccination of Japanese female adolescents. A response rate greater than 60% in each stage of the survey ensures a certain degree of generalizability.

There may be some criticism that our survey did not accurately focus on unvaccinated girls with diverse symptoms in “a multilayered way”, since one eligibility criteria for reported patients was “having at least one of the symptoms”. However, even girls with a single symptom have been reported to the MHLW as “not recovered” cases of adverse events following HPV vaccination.^17^ We also found that both 6-month period prevalence and annual incidence among unvaccinated girls with ≥10 symptoms were “not zero” when number of symptoms was considered as a surrogate variable of multilayered symptoms.

In addition to the prevalence of diverse symptoms among girls without a history of HPV vaccination (Group A), we estimated the prevalence among vaccinated girls whose symptoms occurred after vaccination (Group C). However, these estimates cannot be directly compared between groups because suspension of the proactive recommendation for HPV vaccination in Japan led to a smaller vaccinated population among girls aged 12–14 years (Table 5). Some unstable age-specific prevalence due to a sparse denominator (eg, for girls aged 14 years in Group C) prevented us from obtaining reliable age-standardized estimates. Furthermore, there were many potential biases that could have led to underestimation or overestimation of prevalence in each group (Table 6). Nonetheless, it is noteworthy that the prevalence in Group A seemed to be underestimated overall because situation number 1 in Table 6 (ie, if patients already have diverse symptoms, they will not receive HPV vaccine), which results in overestimation of prevalence, would be very unlikely due to low HPV vaccination coverage at the time of the survey.

**Table 6.** Potential biases in comparing prevalence or distributions of symptoms by HPV vaccination status

Situation number	Process/point	Persons	Potential biases		

*In comparison of prevalence by HPV vaccination status*				*Prevalence among never vaccinated*	*Prevalence among vaccinated*
1	Vaccination	Patients	If patients already have diverse symptoms, they will not receive HPV vaccine.	↑^a^	↓^a^

2	Medical visits	Patients	If patients developed diverse symptoms after HPV vaccination, they are likely to visit the medical institution.	→	↑

3	First-stage survey	Investigators	Since we targeted the patients who visited hospitals, patients who visited clinics were never identified.	↓	↓

4	First-stage survey	Doctors	Doctors are less likely to respond to the survey if they only have experiences in providing medical care for patients with diverse symptoms who have never been vaccinated.	↓	→

5	First-stage survey	Doctors	Doctors are less likely to report patients who have been never vaccinated if the doctors wrongly understand that this survey targets only those patients who have been vaccinated.	↓	→

6	First-stage survey	Doctors	Doctors are less likely to report patients who have been vaccinated if the doctors do not agree with the purpose of the survey.	→	↓^b^

7	Second-stage survey	Doctors	Doctors are less likely to respond to the survey if they only have experiences in providing medical care for patients with diverse symptoms who have never been vaccinated.	↓	→

8	Second-stage survey	Doctors/ Investigators	Patients are categorized as “vaccinated” irrespective of duration from vaccination to symptom onset.	→	↑

9	Second-stage survey	Doctors	Doctors are likely to make decisions as “the symptoms cannot be explained medically” or “the symptoms can be explained by HPV vaccine-related diagnosis”, if the patients with vaccination history visit them.	→	↑

10	Second-stage survey	Doctors	Doctors are likely to report “HPV vaccination history of the patient is unknown” in the case of no definite information on vaccination history in medical records, even if the patients are thought to be unvaccinated, because this survey asks for information to be extracted from already-existing medical data.	↓	→

11	Second-stage survey	Doctors	Doctors are likely to report “the date of HPV vaccination of the patient is unknown” in the case of no definite information on the date in medical records, even if the patient’s symptoms are thought to have occurred after vaccination.	→	↓

*In comparison of distributions of symptoms by HPV vaccination status*				*Frequency of symptoms among never vaccinated*	*Frequency of symptoms among vaccinated*

12	Second-stage survey	Patients	Patients who have history of vaccination are likely to pay attention to symptoms that have been reported to be associated with the vaccine.	→	↑

13	Second-stage survey	Doctors	Doctors are likely to extract information related to symptoms that are thought to be associated with vaccine when they examine patients who have a history of vaccination.	↓	↑

↓: Underestimated, ↑: Overestimated, →: Not affected, HPV, human papillomavirus.

^a^The bias is negligible if HPV vaccination rate is very low.

^b^The patients may be reported by another doctor if they visit several medical institutions at the same time.

There are other reasons why we cannot compare prevalence between unvaccinated and vaccinated girls. First, there were 604 girls with diverse symptoms in Group E whose vaccination status was unknown (Table 5) because information collection in the second-stage survey relied on pre-exiting medical charts. In a hypothesized situation in which all patients in Group E were “never vaccinated” (ie, vaccine coverage: 0%), period prevalence in Group A was greater than that in Group C (eFigure 3). This situation may be plausible when considering the similar characteristics between Groups A and E in the second-stage survey (eFigure 2). Second, patients in Group C included girls whose diverse symptoms occurred more than several months after last HPV vaccination (maximum: 48 months in the second-stage survey). When we limited Group C to those who had shorter durations, the period prevalence was lower than that in Group A (eTable 7). The proportion/number of each symptom between groups in the second-stage survey (eFigure 2) also cannot be compared due to potential bias, as shown in Table 6. Despite these biases, we believe that providing proportions/numbers, with careful interpretation, is meaningful for a better understanding of HPV vaccine issues in Japan.

Reports similar to ours have not yet been published in terms of severity of diverse symptoms. The previous study in Nagoya, Japan, which targeted diverse symptoms among Japanese adolescent girls, differed from our survey not only because it compared the odds of various symptoms in vaccinated and non-vaccinated girls, but also because it evaluated milder symptoms, with cumulative incidences in unvaccinated girls of 0.2% to 25.6%.^16^ While that study showed no association between HPV vaccination and 24 symptoms by comparing vaccinated and unvaccinated girls, another study re-analyzed the data and showed possible associations for several symptoms, such as cognitive impairment and movement disorders.^25^ That study used the same dataset but applied different methodology, in terms of study period, interaction term, age adjustment, and selection of unvaccinated controls. Such discrepancies may indicate the difficulty of controlling confounding and reducing selection bias in the dataset. However, since our study targeted different levels of symptoms, which were more severe and less frequent, findings from that previous study are not directly relevant to our findings.

While our study demonstrated how challenging it was to compare the frequency of diverse symptoms between adolescents with and without HPV vaccination in observational studies, comparability between treatment groups may be retained in pre-licensure clinical trials. eTable 8 summarizes results from phase III, double-blind, randomized controlled trials of HPV vaccines, which included adolescents aged 12–18 years and safety data on serious adverse events during the follow-up period, compared to placebo vaccines or other vaccines comparable to placebo vaccines (eg, hepatitis A virus vaccine) as controls.^26^^–^^36^ Overall, the safety profile was similar between the HPV vaccine and control groups. These reports also consistently showed that the most frequently reported solicited adverse reactions following vaccination were injection site symptoms (pain, redness, and swelling), which were reported significantly more often in the HPV vaccine group than in the control group but were generally transient.

Orthostatic dysregulation and its associated disorders were the most frequent diagnoses and could not be clearly distinguished from diverse symptoms after HPV vaccination. Our findings were in line with the fact that POTS has been frequently evaluated as a possible adverse event following HPV vaccination,^37^^–^^41^ although the majority of reports found no significant safety concern or supportive evidence for a causal relationship. The annual incidence of POTS among unvaccinated girls was estimated to be 6.49 per 100,000 in Finland,^42^ which is consistent with our findings in terms of disease rarity.

The underlying mechanisms of diverse symptoms that can occur among unvaccinated girls are complex and remain incompletely understood. Although generalizability is controversial, some of the recent findings are worth noting. Regarding frequently reported diagnoses in the present survey, pathogenesis for orthostatic dysregulation or POTS may have included impairment of circulatory adjustment against gravitational stress, altered central blood volume, abnormal autonomic reflexes and elevated sympathetic tone, damaged skeletal muscle pump activity, local vascular tension regulation dysfunction, iron insufficiency, mast cell activation, and autoimmune dysfunction.^43^^,^^44^ In patients with somatoform disorders, morphological alterations that encompass motor, limbic, and somatosensory circuits were observed in neuroimaging research.^45^ Although not a frequently reported diagnosis in this study, fibromyalgia can present symptoms similar to diverse symptoms after HPV vaccination because the clinical features of fibromyalgia and persistent somatoform pain disorder or somatization disorder overlap in patients with chronic widespread pain without specific somatic disease factors.^46^ There was a report of an increased inflammatory response in glia cells associated with abnormal brain function in patients with fibromyalgia.^47^ A phase II clinical trial that showed clinical improvement of myalgic encephalopathy/chronic fatigue syndrome after treatment with rituximab suggested that the syndrome may be a variant of an autoimmune disease,^48^ while a subsequent phase III clinical trial failed to detect a significant improvement.^49^ However, considering that a bio-psycho-social model is now widely accepted as the most heuristic approach to chronic pain, it may be impossible to explain diverse symptoms simply via biological mechanisms.^50^

Some limitations in our survey should be mentioned. First, our sampling frame included all hospitals in Japan but did not include clinics because the number of clinics was tenfold or more than that of hospitals. Although severe or difficult-to-diagnose cases are referred to hospitals and patients can visit hospitals directly without being referred by clinics in Japan, our prevalence results among unvaccinated girls remained underestimated. Second, we could not analyze the frequency of diverse symptoms by HPV vaccine type among vaccinated girls because we did not obtain such information during the survey. Third, we could not estimate confidence intervals (CIs) of prevalence. The protocol for a nationwide epidemiological survey on intractable diseases provided a formula to calculate 95% CIs for prevalence.^18^^–^^20^ However, the formula relied on data from the first-stage survey. Since our prevalence was calculated with many parameters from the second-stage survey, estimation of CIs was technically challenging. Epidemiological studies are required to consider the influence of chance, bias, and confounding in a balanced manner. Since our survey was subject to substantial bias in comparing prevalence of diverse symptoms between unvaccinated and vaccinated girls, the highest priority was appropriate interpretation of the prevalence of diverse symptoms among unvaccinated girls. This priority led us to not attempt estimation of CIs, as well as statistical testing, because evaluating the extent of chance would not make sense under the considerable influence of bias.

In conclusion, our nationwide epidemiological survey in Japan revealed that adolescent girls without history of HPV vaccination also visited hospitals with diverse symptoms similar to those following HPV vaccination. We hope our results help explain the complexity of HPV vaccine safety issues in Japan and will provide useful insight into future strategies of signal assessment immediately after detection of AEFI.

## APPENDIX A. SUPPLEMENTARY DATA

eTable 1. Complete list of reported diagnoses of patients whose doctor in charge had responded as “the symptoms are not explicable by the listed diagnoses” (both boys and girls who were reported to the second-stage survey and whose age at onset was ≥12 years, N = 82)

eTable 2. Complete list of reported diagnoses of patients whose doctor in charge had responded as “the symptoms are explicable by the listed diagnoses” and whose diagnosis that was designated as “the most explicable” by the doctor in charge included “due to HPV vaccine” or “after HPV vaccination” (both boys and girls who were reported to the second-stage survey and whose age at onset was ≥12 years, N = 31)

eTable 3. Complete list of reported diagnoses of patients whose doctor in charge had responded as “the symptoms are explicable by the listed diagnoses”, whose diagnosis that was designated as “the most explicable” by the doctor in charge did not include “due to HPV vaccine” or “after HPV vaccination”, and the diagnosis can be clearly distinguished from “diverse symptoms after HPV vaccination (both boys and girls who were reported to the second-stage survey and whose age at onset was ≥12 years, N = 21)

eTable 4. Complete list of reported diagnoses of patients whose doctor in charge had responded as “the symptoms are explicable by the listed diagnoses”, whose diagnosis that was designated as “the most explicable” by the doctor in charge did not include “due to HPV vaccine” or “after HPV vaccination”, and the diagnosis cannot be clearly distinguished from “diverse symptoms after HPV vaccination (both boys and girls who were reported to the second-stage survey and whose age at onset was ≥12 years, N = 435)

eTable 5. Complete list of reported diagnoses of patients whose doctor in charge had responded as “not known whether the symptoms are explicable by the listed diagnosis” (both boys and girls who were reported to the second-stage survey and whose age at onset was ≥12 years, N = 25)

eTable 6. Results of the first-stage survey by departments and hospital characteristics

eTable 7. Estimated period prevalence (per 100,000 population) during the period of July to December 2015 among girls with diverse symptoms, according to duration from last vaccination to symptom onset

eTable 8. Summary results of safety data on serious adverse events from phase III, double-blind, randomized controlled trials of HPV vaccine which included adolescents aged 12–18 years and used placebo vaccines or other vaccines comparable to placebo vaccines (e.g., hepatitis A virus vaccine) as controls

eFigure 1. Number of patients reported to the second-stage survey, and flow diagram of subjects for the analyses.

eFigure 2. Characteristics of patients reported to the second-stage survey. Analyses were limited to girls with diverse symptoms whose age at onset was ≥12 years

eFigure 3. Estimated period prevalence (per 100,000) during the period of July to December 2015 among girls with diverse symptoms in Group A (never vaccinated) and Group C (vaccinated, symptom occurred after vaccination): (i) shows reproduced figures from Table 5, ignoring patients in Group E (unknown vaccination status); (ii) and (iii) show hypothesized situations in which all patients in Group E were (ii) “never vaccinated” (i.e., vaccine coverage: 0%) and (iii) “vaccinated, symptom occurred after vaccination” (i.e., vaccine coverage: 100%).

eMaterials 1. Survey explanation form

eMaterials 2. Decision process whether nor not the reported symptoms correspond to “diverse symptoms after human papillomavirus (HPV) vaccination” (detailed explanation of Table 2)
