# Supplementary material for: A Nationwide Epidemiological Survey of Adolescent Patients With Diverse Symptoms Similar to Those Following Human Papillomavirus Vaccination: Background Prevalence and Incidence for Considering Vaccine Safety in Japan
Source: J Epidemiol. 2022 Jan 5;32(1):34–43. doi: 10.2188/jea.JE20210277 (PMC8666311; doi:10.2188/jea.JE20210277)
Supplement: Supplementary file 1 [file je-32-034-s001.pdf]

eTable 1. Complete list of reported diagnoses of patients whose doctor in charge had responded as "the symptoms are not explicable by the listed diagnoses" (both boys and girls who were reported to the second-stage survey and whose age at onset was ≥12 years, N=82)

Primary diagnoses that were designated by the doctor in charge	Number of patients
Orthostatic dysregulation, orthostatic adjustment disorder, orthostatic hypotension	12
Headache	8
Peripheral neuropathic pain, neuropathic pain	6
Epilepsy	3
Adjustment disorder, truancy	3
Anxiety disorder	3
Bronchial asthma	3
Fibromyalgia	2
Hypotension	2
Somatoform disorder	2
(Others)	
Attention deficit hyperactivity disorder, systemic lupus erythematosus, atopic dermatitis, allergic conjunctivitis, depression, myoclonus, right hand tremor, old rupture of lateral ligament of the right ankle, suspected right knee injury, pain in the right thumb, irritable bowel syndrome, memory impairment, thoracolumbar scoliosis, myofascial pain syndrome, muscle weakness, difficulty in oral intake, cervical sprain, dysmenorrhea, unexplained disease of the extremities, traumatic injury (no fracture), lumbar spinal canal stenosis, left leg neuropathic pain, left arm neuropathic pain, fracture of left sacral spine and transverse process, left radial nerve palsy, prolonged cough, appendicitis, suspected low-tone hearing loss, schizophrenia, head injury, symptoms after surgery for idiopathic scoliosis, refractory pain, complex regional pain syndrome, chronic fatigue syndrome, bilateral knee arthropathy, autoimmune encephalopathy (due to HPV vaccine), suspected vaccination adverse event, none	38
Total	82

HPV, human papillomavirus.

eTable 2. Complete list of reported diagnoses of patients whose doctor in charge had responded as “the symptoms are explicable by the listed diagnoses” and whose diagnosis that was designated as “the most explicable” by the doctor in charge included “due to HPV vaccine” or “after HPV vaccination” (both boys and girls who were reported to the second-stage survey and whose age at onset was ≥12 years, N=31)

Diagnosis designated by the doctor in charge as “the most likely explanation for the observed symptoms”	Number of patients
Autoimmune encephalopathy (due to HPV vaccine)	19
HPV vaccine-associated neuroimmunopathic syndrome (HANS)	3
HPV vaccine-related neuropathy	2
Autoimmune autonomic neuropathy (after HPV vaccination)	1
Autonomic neuropathy (due to HPV vaccine)	1
HPV vaccine-related headache	1
Migraine (due to HPV vaccine)	1
Orthostatic dysregulation (due to HPV vaccine)	1
Chronic fatigue syndrome (after HPV vaccination)	1
Narcolepsy (after HPV vaccination)	1
Total	31

HPV, human papillomavirus.

eTable 3. Complete list of reported diagnoses of patients whose doctor in charge had responded as “the symptoms are explicable by the listed diagnoses”, whose diagnosis that was designated as “the most explicable” by the doctor in charge did not include “due to HPV vaccine” or “after HPV vaccination”, and the diagnosis can be clearly distinguished from “diverse symptoms after HPV vaccination (both boys and girls who were reported to the second-stage survey and whose age at onset was ≥12 years, N=21)

Diagnosis designated by the doctor in charge as “the most likely explanation for the observed symptoms”	Number of patients
Systemic lupus erythematosus	2
Epilepsy	2
Rheumatoid arthritis	1
Cytomegalovirus infection	1
Helicobacter pylori infection	1
Herpes zoster viral myelitis	1
Atlanto-axial dislocation	1
Thoracic spinal cord tumor	1
Primary undifferentiated sarcoma in the pelvis	1
Adult-onset Still’s disease	1
Sudden sensorineural hearing loss	1
Polycystic ovary syndrome (ovarian dysfunction)	1
Crohn’s disease	1
Gastric ulcer	1
Left arm plexus injury	1
Cerebral cavernous angioma	1
Acute hemorrhagic cystitis	1
Postoperative fifth lumbar spondylolisthesis	1
Left knee anterior cruciate injury	1
Total	21

HPV, human papillomavirus.

eTable 4. Complete list of reported diagnoses of patients whose doctor in charge had responded as "the symptoms are explicable by the listed diagnoses", whose diagnosis that was designated as "the most explicable" by the doctor in charge did not include "due to HPV vaccine" or "after HPV vaccination", and the diagnosis cannot be clearly distinguished from "diverse symptoms after HPV vaccination (both boys and girls who were reported to the second-stage survey and whose age at onset was ≥12 years, N=435)

Diagnosis designated by the doctor in charge as "the most likely explanation for the observed symptoms"	Number of patients
Orthostatic dysregulation, orthostatic adjustment disorder, orthostatic hypotension, postural tachycardia syndrome	116
Adjustment disorder, truancy, malaise, abusive behavior, immature personality	46
Somatoform disorder, somatization disorder, somatoform autonomic dysfunction	39
Dissociative disorder, dissociative movement disorder, dissociative spasms, conversion disorder	27
Depression, depressive state, depressed state, adolescent depression, childhood depression, mild depressive episode	25
Psychosomatic disorder, psychogenic response, psychogenic visual impairment, psychogenic astasia, psychogenic abdominal pain, psychogenic pain, neurosis	25
Anxiety disorder, anxiety neurosis, acute stress reaction, severe stress reaction, panic disorder, post-traumatic stress disorder	21
Developmental disability, some developmental bias, autism spectrum disorder, pervasive developmental disorder, Asperger's syndrome	20
Headache, migraine, tension headache	20
Irritable bowel syndrome	14
Sleep disorder, insomnia, periodic somnolence, sleep-wake rhythm disturbance	10
Schizophrenia, bipolar disorder, bromhidrosis	6
Juvenile fibromyalgia, juvenile idiopathic arthritis	5
Dysautonomia, autonomic dysfunction	4
(Others)<br>Autoimmune autonomic ganglionopathy, autoimmune encephalopathy, functional motor disorder (symptom), systemic dystonia, low cerebrospinal fluid syndrome, cerebrospinal fluid leak, cerebral oedema, head trauma sequelae, involuntary movements, paroxysmal kinesigenic choreoathetosis, complex regional pain syndrome, right	57

lower limb complex regional pain syndrome, lower limb disuse syndrome, chronic fatigue syndrome, abdominal pain, gastrointestinal motility disorder, intercostal neuralgia, traumatic cervical syndrome, numbness of extremities on the left side, persistent pain, amnestic syndrome, eosinophilic gastroenteritis, low back pain, anorexia nervosa, etc.	
Total	435

HPV, human papillomavirus.

eTable 5. Complete list of reported diagnoses of patients whose doctor in charge had responded as “not known whether the symptoms are explicable by the listed diagnosis” (both boys and girls who were reported to the second-stage survey and whose age at onset was ≥12 years, N=25)

Primary diagnoses that were designated by the doctor in charge	Number of patients
Somatoform disorder, suspected somatoform disorder, persistent somatoform pain disorder	4
Truancy, adjustment disorder	3
Depression, suspected depression	2
Suspected autism spectrum disorder	1
Suspected orthostatic dysregulation	1
HPV vaccine-related neuropathy	1
Left peroneal nerve palsy	1
Tension headache	1
Pain disorder	1
Complex regional pain syndrome	1
Multiple sclerosis	1
Cerebellar ataxia	1
None listed	7
Total	25

HPV, human papillomavirus.

eTable 6. Results of the first-stage survey by departments and hospital characteristics

Departments	Strata	Number of departments					Reported number of patients	
		Eligible	Sampled (% [a])		Responded (% [b])		Boys	Girls
Pediatrics	University hospitals	119	119	(100.0)	94	(79.0)	67	142
	≥500 beds	226	226	(100.0)	175	(77.4)	40	46
	400–499 beds	223	223	(100.0)	176	(78.9)	45	58
	300–399 beds	340	340	(100.0)	245	(72.1)	53	123
	200–299 beds	323	323	(100.0)	232	(71.8)	12	50
	100–199 beds	641	308	(48.0)	190	(61.7)	25	26
	<99 beds	724	356	(49.2)	182	(51.1)	1	1
	Subtotal	2,596	1,895	(73.0)	1,294	(68.3)	243	446
Neurology	University hospitals	127	127	(100.0)	94	(74.0)	26	79
	≥500 beds	207	207	(100.0)	140	(67.6)	3	31
	400–499 beds	177	177	(100.0)	108	(61.0)	6	17
	300–399 beds	274	274	(100.0)	159	(58.0)	0	14
	200–299 beds	294	294	(100.0)	154	(52.4)	5	12
	100–199 beds	611	303	(49.6)	167	(55.1)	3	3
	<99 beds	299	147	(49.2)	74	(50.3)	16	8
	Subtotal	1,989	1,529	(76.9)	896	(58.6)	59	164
Anesthesiology (pain clinic)	University hospitals	83	83	(100.0)	70	(84.3)	13	58
	≥500 beds	68	68	(100.0)	57	(83.8)	3	6
	400–499 beds	40	40	(100.0)	27	(67.5)	1	3
	300–399 beds	48	48	(100.0)	37	(77.1)	0	2
	200–299 beds	22	22	(100.0)	20	(90.9)	0	2
	100–199 beds	35	18	(51.4)	13	(72.2)	1	1
	<99 beds	23	12	(52.2)	6	(50.0)	0	1
	Subtotal	319	291	(91.2)	230	(79.0)	18	73
Obstetrics and gynecology	University hospitals	95	95	(100.0)	79	(83.2)	0	2
	≥500 beds	228	228	(100.0)	176	(77.2)	0	2
	400–499 beds	213	213	(100.0)	161	(75.6)	0	2
	300–399 beds	316	316	(100.0)	220	(69.6)	0	4
	200–299 beds	270	270	(100.0)	172	(63.7)	0	0

	100–199 beds	397	193	(48.6)	121	(62.7)	0	4
	<99 beds	342	171	(50.0)	108	(63.2)	0	2
	Subtotal	1,861	1,486	(79.8)	1,037	(69.8)	0	16
Orthopedic surgery	University hospitals	129	129	(100.0)	103	(79.8)	13	23
	≥500 beds	261	261	(100.0)	177	(67.8)	13	15
	400–499 beds	254	254	(100.0)	173	(68.1)	7	13
	300–399 beds	429	429	(100.0)	288	(67.1)	2	3
	200–299 beds	535	535	(100.0)	322	(60.2)	14	9
	100–199 beds	1,577	786	(49.8)	482	(61.3)	13	15
	<99 beds	1,595	792	(49.7)	404	(51.0)	15	9
	Subtotal	4,780	3,186	(66.7)	1,949	(61.2)	77	87
Gastroenterology	University hospitals	139	139	(100.0)	102	(73.4)	1	2
	≥500 beds	311	311	(100.0)	177	(56.9)	1	1
	400–499 beds	348	348	(100.0)	196	(56.3)	0	1
	300–399 beds	619	619	(100.0)	312	(50.4)	6	27
	200–299 beds	937	937	(100.0)	486	(51.9)	8	19
	100–199 beds	2,438	1,211	(49.7)	671	(55.4)	2	1
	<99 beds	2,671	1,326	(49.6)	772	(58.2)	22	9
	Subtotal	7,463	4,891	(65.5)	2,716	(55.5)	40	60
Rheumatology	University hospitals	120	120	(100.0)	95	(79.2)	7	20
	≥500 beds	77	77	(100.0)	58	(75.3)	1	3
	400–499 beds	64	64	(100.0)	38	(59.4)	1	5
	300–399 beds	85	85	(100.0)	41	(48.2)	0	0
	200–299 beds	122	122	(100.0)	63	(51.6)	0	1
	100–199 beds	315	158	(50.2)	98	(62.0)	2	4
	<99 beds	298	148	(49.7)	76	(51.4)	0	0
	Subtotal	1,081	774	(71.6)	469	(60.6)	11	33
General practice	University hospitals	85	85	(100.0)	56	(65.9)	3	9
	≥500 beds	76	76	(100.0)	42	(55.3)	0	0
	400–499 beds	37	37	(100.0)	20	(54.1)	0	1
	300–399 beds	43	43	(100.0)	24	(55.8)	0	1
	200–299 beds	19	19	(100.0)	8	(42.1)	0	0
	100–199 beds	51	26	(51.0)	15	(57.7)	2	1
	<99 beds	15	7	(46.7)	3	(42.9)	0	0

	Subtotal	326	293	(89.9)	168	(57.3)	5	12
Neurosurgery	University hospitals	125	125	(100.0)	95	(76.0)	21	21
	≥500 beds	237	237	(100.0)	157	(66.2)	9	6
	400–499 beds	214	214	(100.0)	144	(67.3)	0	2
	300–399 beds	339	339	(100.0)	206	(60.8)	9	3
	200–299 beds	345	345	(100.0)	203	(58.8)	12	8
	100–199 beds	677	336	(49.6)	168	(50.0)	2	8
	<99 beds	416	207	(49.8)	99	(47.8)	0	0
	Subtotal	2,353	1,803	(76.6)	1,072	(59.5)	53	48
Psychiatry/psychosomatic medicine	University hospitals	128	128	(100.0)	91	(71.1)	196	277
	≥500 beds	256	256	(100.0)	144	(56.3)	19	58
	400–499 beds	258	258	(100.0)	145	(56.2)	51	68
	300–399 beds	381	381	(100.0)	194	(50.9)	26	49
	200–299 beds	562	562	(100.0)	287	(51.1)	51	78
	100–199 beds	747	371	(49.7)	202	(54.4)	18	20
	<99 beds	225	110	(48.9)	58	(52.7)	3	2
	Subtotal	2,557	2,066	(80.8)	1,121	(54.3)	364	552
Special departments	—	88	88	(100.0)	85	(96.6)	33	161
Total	University hospitals	1,150	1,150	(100.0)	879	(76.4)	347	633
	≥500 beds	1,947	1,947	(100.0)	1,303	(66.9)	89	168
	400–499 beds	1,828	1,828	(100.0)	1,188	(65.0)	111	170
	300–399 beds	2,874	2,874	(100.0)	1,726	(60.1)	96	226
	200–299 beds	3,429	3,429	(100.0)	1,947	(56.8)	102	179
	100–199 beds	7,489	3,710	(49.5)	2,127	(57.3)	68	83
	<99 beds	6,608	3,276	(49.6)	1,782	(54.4)	57	32
	Special hospitals	88	88	(100.0)	85	(96.6)	33	161
	Total	25,413	18,302	(72.0)	11,037	(60.3)	903	1,652

[a] Number of sampled/number of eligible.

[b] Number of responded/number of sampled.

eTable 7. Estimated period prevalence (per 100,000 population) during the period of July to December 2015 among girls with diverse symptoms, according to duration from last vaccination to symptom onset

	Duration from last vaccination to symptom	Proportion among female patients with diverse symptoms [a]	Period prevalence among girls aged 12–18 years (per 100,000)
Vaccinated, symptoms occurred after vaccination	No consideration	1.00	27.8
	≤1 year	0.63	17.5
	≤6 months	0.55	15.3
	≤3 months	0.41	11.4
	≤1 month	0.31	8.6
Never vaccinated	—	—	20.2

HPV, human papillomavirus.

[a] Calculated using information from the second-stage survey.

eTable 8. Summary results of safety data on serious adverse events from phase III, double-blind, randomized controlled trials of HPV vaccine which included adolescents aged 12–18 years and used placebo vaccines or other vaccines comparable to placebo vaccines (e.g., hepatitis A virus vaccine) as controls

First Author [Ref number]	Year of study initiation / Country	Subjects (age)	HPV vaccine	Control vaccine	Follow-up time	Results of safety analysis (comparisons are presented as HPV vaccine group vs. control vaccine group)
The FUTURE II Study Group [26]	2002 / 13 countries in Asia-Pacific, North America, Latin America, and Europe	12,167 women (15–26 years)	qHPV vaccine (n=6,087)	Aluminum-containing placebo (n=6,080)	Mean: 3 years	Number of subjects with follow-up data: 6019 for HPV vaccine group and 6031 for placebo group<br><br>Subjects with any serious event: 45 (0.7%) vs. 54 (0.9%); RD −0.1% [−0.5% to 0.2%]<br>Subjects with serious injection-related event §: 3 (<0.1%) vs. 2 (<0.1%); RD 0% [−0.1% to 0.1%]<br>Discontinuation due to serious adverse event: 7 (0.1%) vs. 6 (0.1%); RD 0% [−0.1% to 0.2%]<br>Discontinuation due to serious injection-related event: 0 vs. 1 (<0.1%); RD 0% [−0.1% to 0.1%]<br>Discontinuation due to death ¶: 7 (0.1%) vs. 5 (0.1%); RD 0% [−0.1% to 0.2%]<br><br>"§ In the HPV vaccine group, the serious adverse events were gastroenteritis, headache, hypertension, injection-site pain, and a decrease in joint movement at the injection site. In the placebo group, the serious adverse events were hypersensitivity to the injection (for which one subject discontinued participation in the study), chills, headache, and fever.<br>¶ Causes of death in the HPV vaccine group were pneumonia and sepsis, overdose of an illicit drug, traffic accident (three subjects), pulmonary embolism, and infective thrombosis. The causes of death in the placebo group were suicide (two subjects), asphyxia, and traffic accident (two subjects). None of the deaths were judged by the research investigator to be related to HPV vaccine or placebo."

						Specific serious adverse experiences by organ system	
						Blood and lymphatic system disorders	1 (0.0%) vs. 0 (0.0%); RD 0.0% [-0.1% to 0.1%]
						Cardiac disorders	2 (0.0%) vs. 1 (0.0%); RD 0.0% [-0.1% to 0.1%]
						Gastrointestinal disorders	2 (0.0%) vs. 2 (0.0%); RD 0.0% [-0.1% to 0.1%]
						General disorders and administration site conditions	0 (0.0%) vs. 2 (0.0%); RD 0.0% [-0.1% to 0.0%]
						Hepatobiliary disorders	1 (0.0%) vs. 0 (0.0%); RD 0.0% [-0.1% to 0.1%]
						Immune system disorders	0 (0.0%) vs. 2 (0.0%); RD 0.0% [-0.1% to 0.0%]
						Infections and infestations	9 (0.1%) vs. 10 (0.2%); RD 0.0% [-0.2% to 0.1%]
						Injury, poisoning and procedural complications	6 (0.1%) vs. 4 (0.1%); RD 0.0% [-0.1% to 0.2%]
						Musculoskeletal and connective tissue disorders	0 (0.0%) vs. 1 (0.0%); RD 0.0% [-0.1% to 0.1%]
						Neoplasms benign, malignant and unspecified (incl cysts and polyps)	0 (0.0%) vs. 1 (0.0%); RD 0.0% [-0.1% to 0.1%]
						Nervous system disorders	4 (0.1%) vs. 1 (0.0%); RD 0.0% [-0.0% to 0.2%]
						Pregnancy, puerperium and perinatal conditions	19 (0.3%) vs. 26 (0.4%); RD -0.1% [-0.4% to 0.1%]
						Psychiatric disorders	1 (0.0%) vs. 2 (0.0%); RD 0.0% [-0.1% to 0.1%]
						Renal and urinary disorders	0 (0.0%) vs. 1 (0.0%); RD 0.0% [-0.1% to 0.1%]
						Reproductive system and breast disorders	2 (0.0%) vs. 3 (0.0%); RD 0.0% [-0.1% to 0.1%]
						Respiratory, thoracic and mediastinal disorders	2 (0.0%) vs. 3 (0.0%); RD 0.0% [-0.1% to 0.1%]
						Skin and subcutaneous tissue disorders	1 (0.0%) vs. 1 (0.0%); RD 0.0% [-0.1% to 0.1%]
						Vascular disorders	3 (0.0%) vs. 1 (0.0%); RD 0.0% [-0.1% to 0.1%]

Garland SM, FUTURE I Investigators [27]	2002 / 16 countries in Asia–Pacific, North America, Latin America, and Europe	5,455 women (16–24 years)	qHPV vaccine (n=2,723)	Aluminum-containing placebo (n=2,732)	Mean: 3 years	Number of participants assessed: 2673 for HPV vaccine group and 2672 for placebo group<br>Serious event 48 (1.8%) vs. 45 (1.7%); RD 0.1% [-0.6% to 0.8%]<br>Vaccine-related event 1 (<0.1%) vs. 0 (0%); RD 0% [-0.1% to 0.2%]<br>Discontinuation because of event 2 (0.1%) vs. 3 (0.1%); RD 0% [-0.3% to 0.2%]<br>Discontinuation because of vaccine-related event 0 (0%) vs. 0 (0%); RD 0% [-0.2% to 0.2%]<br>Death § 2 (0.1%) vs. 2 (0.1%); RD 0% [-0.2% to 0.2%]<br>"§ There were two deaths in the HPV vaccine group, one as a result of a car accident 342 days after administration of the third dose, and one by suicide 1373 days after enrollment. There were two deaths in the placebo group, one from deep-vein thrombosis, renal insufficiency, and shock to the lung 204 days after administration of the third dose, and one as a result of a traffic accident 1 day after administration of the second dose. None of these deaths were considered by the investigator to be related to the HPV vaccine or placebo."<br><br>Specific serious adverse experiences by organ system<br>Blood and lymphatic system disorders 1 (0.0%) vs. 0 (0.0%); RD 0.0% [-0.1% to 0.2%]<br>Hepatobiliary disorders 1 (0.0%) vs. 0 (0.0%); RD 0.0% [-0.1% to 0.2%]<br>Infections and infestations 10 (0.4%) vs. 2 (0.1%); RD 0.3% [0.0% to 0.6%]<br>Injury, poisoning and procedural complications 19 (0.7%) vs. 27 (1.0%); RD -0.3% [-0.8% to 0.2%]<br>Musculoskeletal and connective tissue disorders 0 (0.0%) vs. 1 (0.0%); RD 0.0% [-0.2% to 0.1%]<br>Nervous system disorders 1 (0.0%) vs. 4 (0.1%); RD -0.1% [-0.4% to 0.1%]<br>Pregnancy, puerperium and perinatal conditions 14 (0.5%) vs. 11 (0.4%); RD 0.1% [-0.3% to 0.5%]<br>Psychiatric disorders 1 (0.0%) vs. 0 (0.0%); RD 0.0% [-0.1% to 0.2%]<br>Renal and urinary disorders 0 (0.0%) vs. 1 (0.0%); RD 0.0% [-0.2% to 0.1%]<br>Reproductive system and breast disorders 0 (0.0%) vs. 1 (0.0%); RD 0.0% [-0.2% to 0.1%]<br>Respiratory, thoracic and mediastinal disorders 3 (0.1%) vs. 1 (0.0%); RD 0.1% [-0.1% to 0.3%]<br>Vascular disorders 1 (0.0%) vs. 1 (0.0%); RD 0.0% [-0.2% to 0.2%]

Tay EH [28]	2002 / Countries in Asia-Pacific region including those participating in FUTURE I and FUTURE II study	814 females (16–26 years)	qHPV vaccine (n=461)	Aluminum-containing placebo (n=353)	Median: 3 years	Number of participants assessed: 453 for HPV vaccine group and 347 for placebo group<br><br>Serious adverse event: 11 (2.4%) vs. 3 (0.9%) (P=0.095)<br>"A case of gastroenteritis in the HPV vaccine group, with onset within 5 days of dose 2, was considered possibly vaccine related. Two deaths occurred among HPV vaccine recipients with serious adverse events, a suicide 1177 days after dose 3 and pneumonia with sepsis beginning 625 days after dose 3. These deaths were determined by the investigators as not vaccine related. The remaining serious adverse events among HPV vaccine recipients, all of which were individual cases ending in recovery and considered probably or definitely not vaccine related, were as follows (with timing of onset in parentheses): reflux esophagitis (2 days after dose 1), gastroenteritis (13 days after dose 3), pelvic inflammatory disease (1 day after dose 2), postoperative infection (785 days after dose 3), streptococcal tonsillitis (7 days after dose 1), polytraumatism (10 days after dose 1), breech presentation (261 days after dose 2), and hyperventilation (15 days after dose 1). The 3 serious adverse events among placebo recipients were postprocedural hemorrhage (in 2 participants, 575 and 1059 days after dose 3) and dizziness (in 1 participant, 44 days after dose 2)."
Paavonen J, HPV PATRICIA Study Group [29]	2004 / 14 countries in Asia Pacific, Europe, Latin America, and North America (Australia, Belgium, Brazil, Canada, Finland, Germany, Italy, Mexico, Philippines, Spain, Taiwan, Thailand, UK, and USA)	18,644 females (15–25 years)	bHPV vaccine (n=9,319)	Hepatitis A virus vaccine (n=9,325)	Mean: 34.9 months	Serious adverse event 701 (8%) vs. 699 (8%)<br>Vaccine-related serious adverse event 11 (<1%) vs. 6 (<1%)<br>Medically significant condition 2960 (32%) vs. 3025 (32%)<br>New-onset chronic disease 251 (3%) vs. 268 (3%)<br>New-onset autoimmune disease 78 (<1%) vs. 77 (<1%)<br>Deaths‡ 9 (<1%) vs. 8 (<1%)<br><br>"‡No deaths were thought to be possibly related to vaccination in either group."

Block SL [30]	(Data from 5 phase IIb/III clinical trials including FUTURE I and FUTURE II study)	21,464 females (9–26 years) and males (9–16 years)	qHPV vaccine (n=11,778)	Aluminum-containing or nonaluminum-containing (saline) placebo (n=9,686)	Median: 3.6 years	Number of subjects with follow-up data: 11641 for HPV vaccine group and 9578 for placebo group	
						Serious Adverse Events by System Organ Class	
						Blood/lymphatic system	3 (0.03%) vs. 0 (0.0%)
						Cardiac	3 (0.03%) vs. 1 (0.01%)
						Gastrointestinal	4 (0.03%) vs. 2 (0.02%)
						General	0 (0.0%) vs. 2 (0.02%)
						Hepatobiliary	2 (0.02%) vs. 0 (0.0%)
						Immune system	0 (0.0%) vs. 2 (0.02%)
						Infections/infestations	22 (0.2%) vs. 14 (0.1%)
						Injury/poisoning/procedural	26 (0.2%) vs. 32 (0.3%)
						Metabolism/nutrition	2 (0.02%) vs. 0 (0.0%)
						Musculoskeletal/connective tissue	1 (0.01%) vs. 2 (0.02%)
						Neoplasms benign malignant, unspecified (including cysts and polyps)	1 (0.01%) vs. 1 (0.01%)
						Nervous system	5 (0.04%) vs. 5 (0.05%)
						Pregnancy/puerperium/perinatal	34 (0.3%) vs. 38 (0.4%)
						Psychiatric	3 (0.03%) vs. 2 (0.02%)
						Renal/urinary	2 (0.02%) vs. 2 (0.02%)
						Reproductive system/breast	4 (0.03%) vs. 4 (0.04%)
						Respiratory/thoracic/mediastinal	5 (0.04%) vs. 4 (0.04%)
						Skin/subcutaneous tissue	1 (0.01%) vs. 1 (0.01%)
						Vascular	4 (0.03%) vs. 2 (0.02%)

						Total number of subjects with a serious systemic adverse event 107 (0.9%) vs. 103 (1.1%)<br>Total number of subjects with a serious injection site adverse event 1 (0.01%) vs. 0 (0.0%)<br><br>“Eighteen study subjects who received ≥1 dose of HPV vaccine (n = 11 [0.1%]) or placebo (n = 7 [0.1%]) died during the 5 studies (note: there were 5 additional deaths reported in ongoing studies that are not included in this report and 1 death that was reported after the completion of Protocol 018). All deaths were classified by the investigator as not related to study treatment/ procedures. Road traffic accidents (n = 7) and suicide (n = 3) accounted for over half of the deaths. The causes of the remaining 8 fatalities were as follows (treatment group/relative day of onset following the last dose): pneumonia/sepsis (HPV vaccine/625 days), pancreatic cancer (HPV vaccine/578 days), infective thrombosis/myocarditis/septic shock (HPV vaccine/359 days), arrhythmia (HPV vaccine/27 days), pulmonary embolism (HPV vaccine/20 days), convulsion/overdose from nonstudy medication (HPV vaccine/4 days), asphyxia (placebo/256 days), and pulmonary embolism (placebo/202 days).”
Medina DM, ; HPV-013 Study Group [31] [a]	2004 / 12 countries (Australia, Colombia, the Czech Republic, France, Germany, Honduras, Korea, Norway, Panama, Spain, Sweden, and Taiwan)	2,067 females (10–14 years)	bHPV vaccine (n=1,035)	Hepatitis A virus vaccine (n=1,032)	Up to 12 months	Serious adverse events<br>Reported up to month 7 11 (1.1%) [0.5% to 1.9%] vs.13 (1.3%) [0.7% to 2.1%]<br>Reported between months 7 to 12 13/1014 (1.3%) [0.7% to 2.2%] vs. 10/1009 (1.0%) [0.5% to 1.8%]<br><br>New onset chronic disease<br>Reported up to month 7 25 (2.4%) [1.6% to 3.5%] vs. 21 (2.0%) [1.3% to 3.1%]<br>Reported between months 7 to 12 3/1014 (0.3%) [0.1% to 0.9%] vs. 6/1009 (0.6%) [0.2% to 1.3%]<br><br>Medically significant condition<br>Reported within 30-days post-vaccination period 130 (12.6%) [10.6% to 14.7%] vs. 160 (15.5%) [13.3% to 17.9%]<br>Reported between months 7 to 12 36/1014 (3.6%) [2.5% to 4.9%] vs. 35/1009 (3.5%) [2.4% to 4.8%]

Kim YJ [32] [a]	2005 / Korea	321 females (10–14 years)	bHPV vaccine (n=160)	Hepatitis A virus vaccine (n=161)	Up to 7 months	Number of participants assessed: 126 for HPV vaccine group, 132 for control vaccine group<br><br>Onset of chronic diseases that were identified during the study<br>Contact dermatitis (two subjects in the HPV vaccine group)<br>Urticaria (two subjects in the HPV vaccine group)<br>Atopic dermatitis (one subject in the HPV vaccine group and two subjects in the control vaccine group)<br><br>"One vaccine recipient in the control vaccine group reported a serious adverse event (gastroenteritis) after the second dose that required hospitalization. This serious adverse event was judged as not being vaccine-related, resolved without sequelae and the subject completed the vaccination course. "<br><br>"The occurrence of medically significant conditions reported during the study period was similar in both groups (HPV vaccine group: 6.9%; control vaccine group: 6.2%)."
Sow PS [33]	2007 / Africa	676 females (10–25 years)	bHPV vaccine (n=450)	Placebo vaccine containing $Al(OH)_3$ alone (n=226)	Up to 12 months	Death 0 (0.0%) [0.0% to 0.8%] vs. 0 (0.0%) [0.0% to 1.6%]<br>Serious adverse event 17 (3.8%) [2.2% to 6.0%] vs. 14 (6.2%) [3.4% to 10.2%]<br>Adverse event leading to premature discontinuation 0 (0.0%) [0.0% to 0.8%] vs. 0 (0.0%) [0.0% to 1.6%]<br>Medically significant condition 312 (69.3%) [64.8% to 73.6%] vs. 170 (75.2%) [69.1% to 80.7%]<br>New onset chronic disease 11 (2.4%) [1.2% to 4.3%] vs. 11 (4.9%) [2.5% to 8.5%]<br>New onset autoimmune disease 2 (0.4%) [0.1% to 1.6%] vs. 2 (0.9%) [0.1% to 3.2%]

Zhu F [34] [b]	2009 / China	750 females (9–17 years)	bHPV vaccine (n=374)	Placebo vaccine containing $Al(OH)_3$ alone (n=376)	Up to 12 months	Serious adverse events 5 (1.3%) [0.4% to 3.1%] vs. 2 (0.5%) [0.1% to 1.9%]<br>Related serious adverse events 0 (0.0%) vs. 0 (0.0%)<br>Medically significant conditions 14 (3.7%) [2.1% to 6.2%] vs. 11 (2.9%) [1.5% to 5.2%]<br>Potential immune-mediated diseases NA vs. NA<br>New onset autoimmune diseases 0 (0.0%) [0.0% to 1.0%] vs. 2 (0.5%) [0.1% to 1.9%]
Zhu FC [35]	2008 / China	6,051 females (18–25 years)	bHPV vaccine (n=3,026)	Placebo vaccine containing $Al(OH)_3$ alone (n=3,025)	Mean: 62 months	Serious adverse event 56 (1.9%) vs. 81 (2.7%)<br>Medically significant condition 186 (6.1%) vs. 185 (6.1%)<br>New onset chronic disease 9 (0.3%) vs. 12 (0.4%)<br>Hyperthyroidism 0 vs. 1 (0.0%)<br>Allergy to arthropod bite 0 vs. 1 (0.0%)<br>Anaphylactic reaction 1 (0.0%) vs. 0<br>Hypersensitivity 1 (0.0%) vs. 1 (0.0%)<br>Arthritis 0 vs. 1 (0.0%)<br>Gestational diabetes 0 vs. 1 (0.0%)<br>Dermatitis allergic 3 (0.1%) vs. 4 (0.1%)<br>Dermatitis atopic 1 (0.0%) vs. 0<br>Dermatitis contact 0 vs. 1 (0.0%)<br>Psoriasis 1 (0.0%) vs. 1 (0.0%)<br>Urticaria 2 (0.1%) vs. 2 (0.1%)<br>New onset autoimmune disease 2 (0.1%) vs. 2 (0.1%)<br>Hyperthyroidism 0 vs. 1 (0.0%)<br>VIIth nerve paralysis 1 (0.0%) vs. 0<br>Psoriasis 1 (0.0%) vs. 1 (0.0%)<br>Deaths* 0 vs. 3

						* "Three women in the control group died, one with a recorded cause of gastric neoplasm and two through suicide. No deaths were considered related to vaccination. No women in the HPV vaccine group died."
Mikamo H [36]	2013 / Japan	1,124 men (16–26 years)	qHPV vaccine (n=562)	Placebo vaccine containing aluminum hydroxyphosphate sulfate adjuvant alone (n=562)	Up to 36 months	"No serious adverse events were reported within 15 days of any vaccination. However, one participant in the placebo group died on Day 440 after completion of the three-dose regimen (suicide); this death was not considered vaccine related. No other serious adverse events were reported over the entire study period."

HPV, human papillomavirus vaccine; qHPV vaccine, quadrivalent human papillomavirus vaccine, *Gardasil/Silgard*®; bHPV vaccine, bivalent human papillomavirus vaccine, *Cervarix*®; RD, risk difference (HPV vaccine group minus control vaccine group); NA, not applicable.

Note: Numbers in Table expressed as n (%) [95% confidence interval], otherwise indicated. Reports on Escherichia coli-produced bivalent HPV vaccine were not included.

[a] The study was "observer-blind", not "double-blind", due to differences in the visual appearance of study vaccines. However, the study was considered to be double-blind because study staff who administered vaccine were not otherwise involved in study conduct.

[b] The paper covered two phase III, randomized controlled trials which targeted girls aged 9–17 years and women aged 26–45 years. Results from a trial targeted girls aged 9–17 years were extracted.

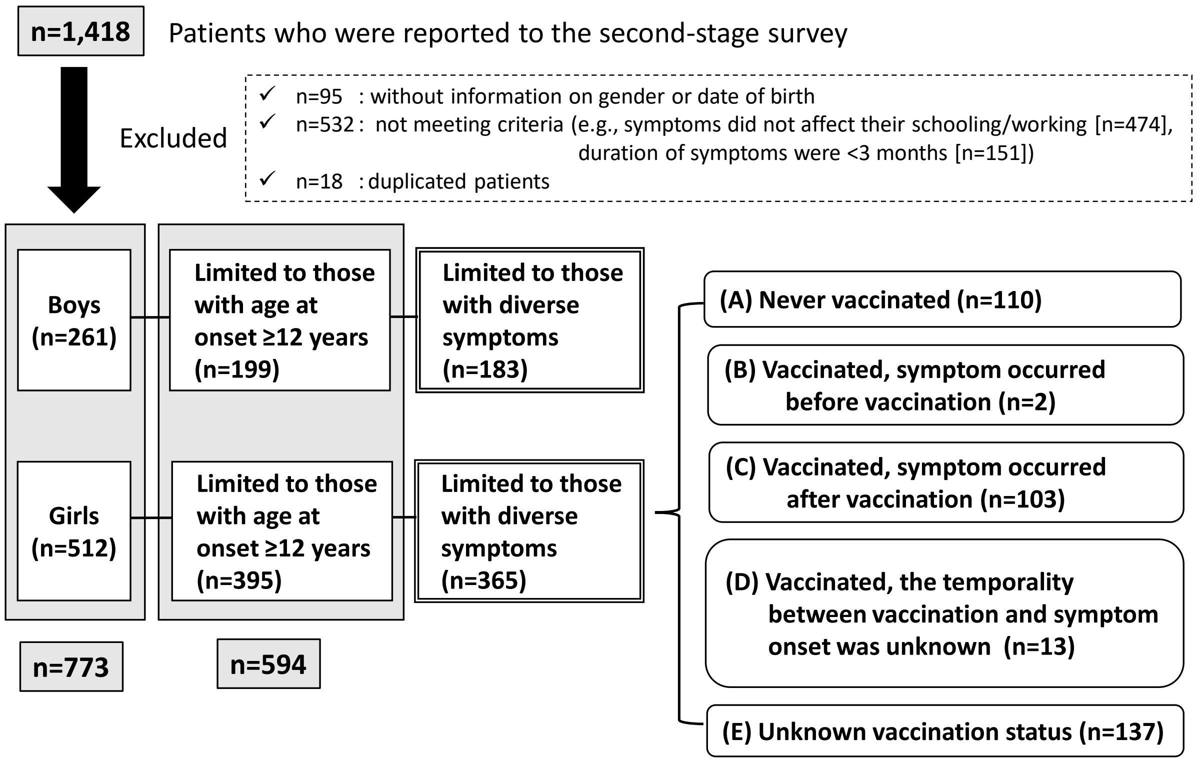

eFigure 1. Number of patients reported to the second-stage survey, and flow diagram of subjects for the analyses.

(i) Pain or sensory dysfunction (light, sound and smell)

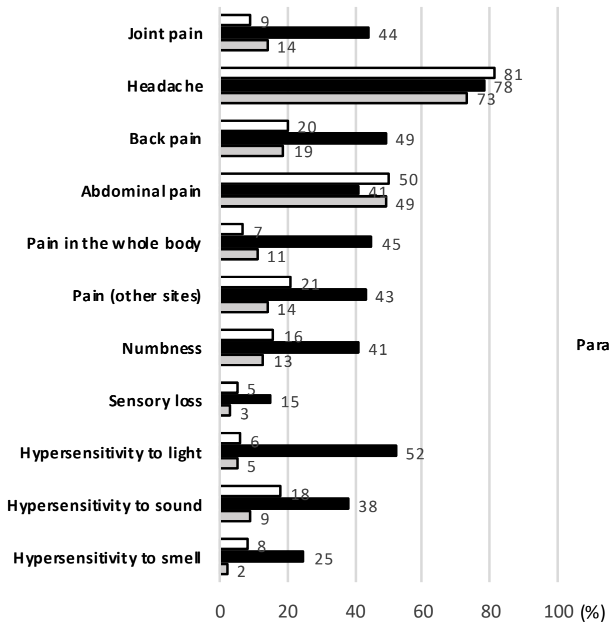

(ii) Motor dysfunction

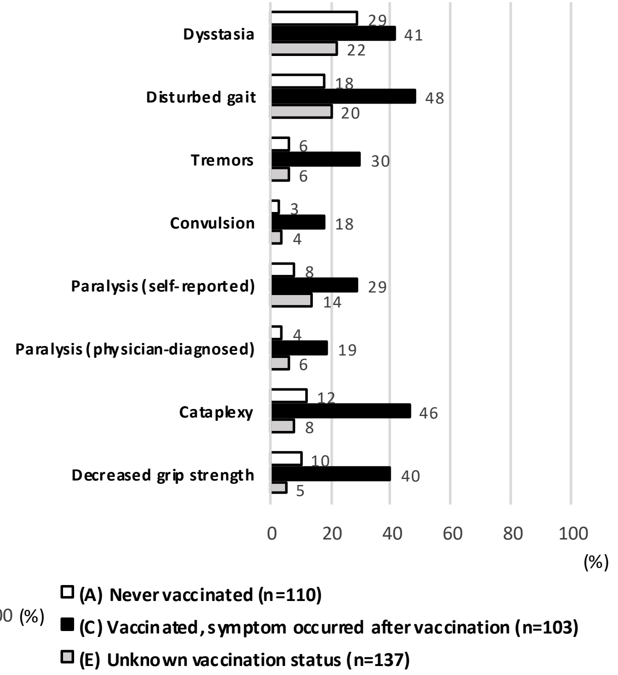

(iii) Autonomic dysfunction

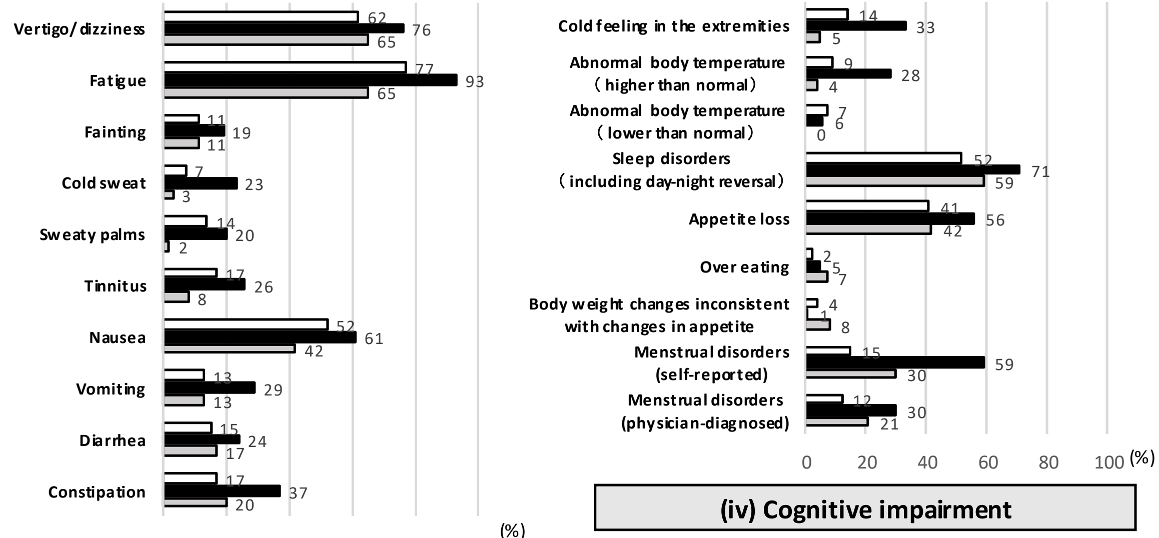

(iv) Cognitive impairment

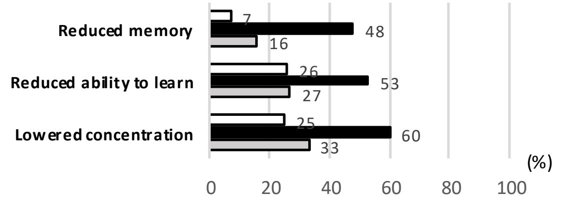

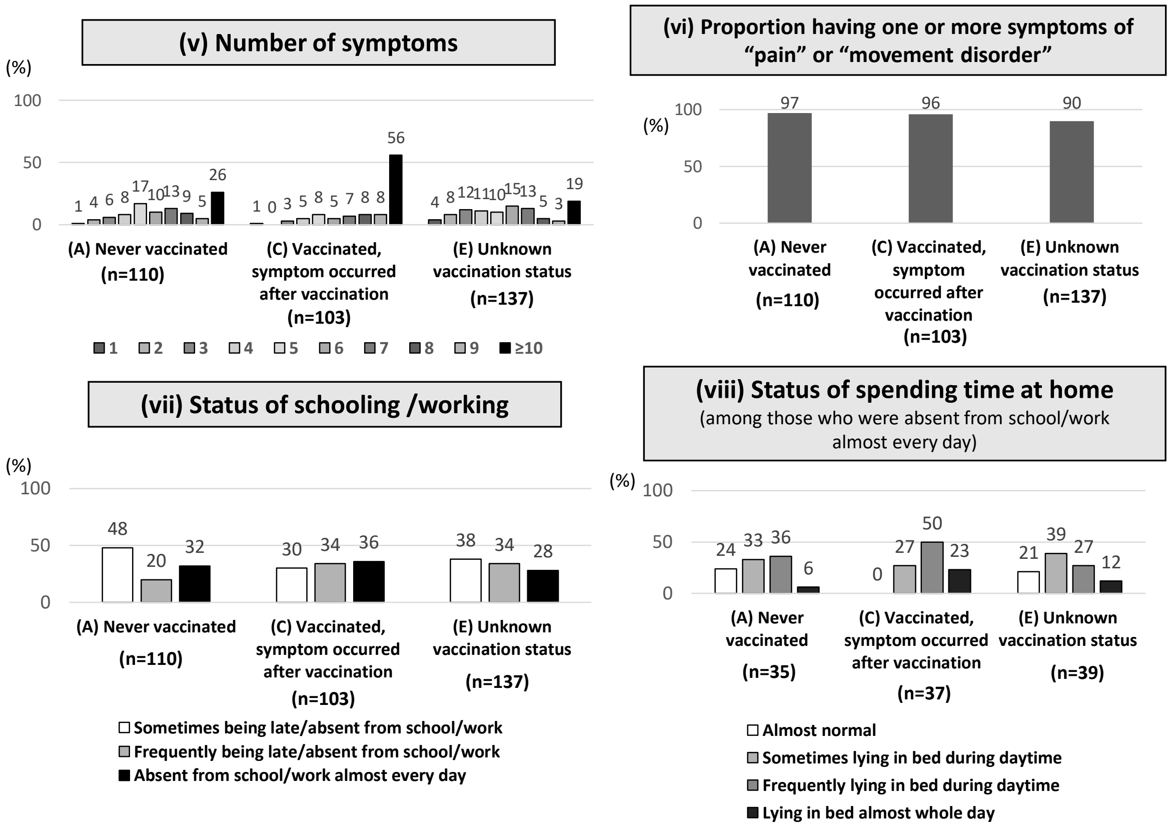

eFigure 2. Characteristics of patients reported to the second-stage survey. Analyses were limited to girls with diverse symptoms whose age at onset was ≥12 years

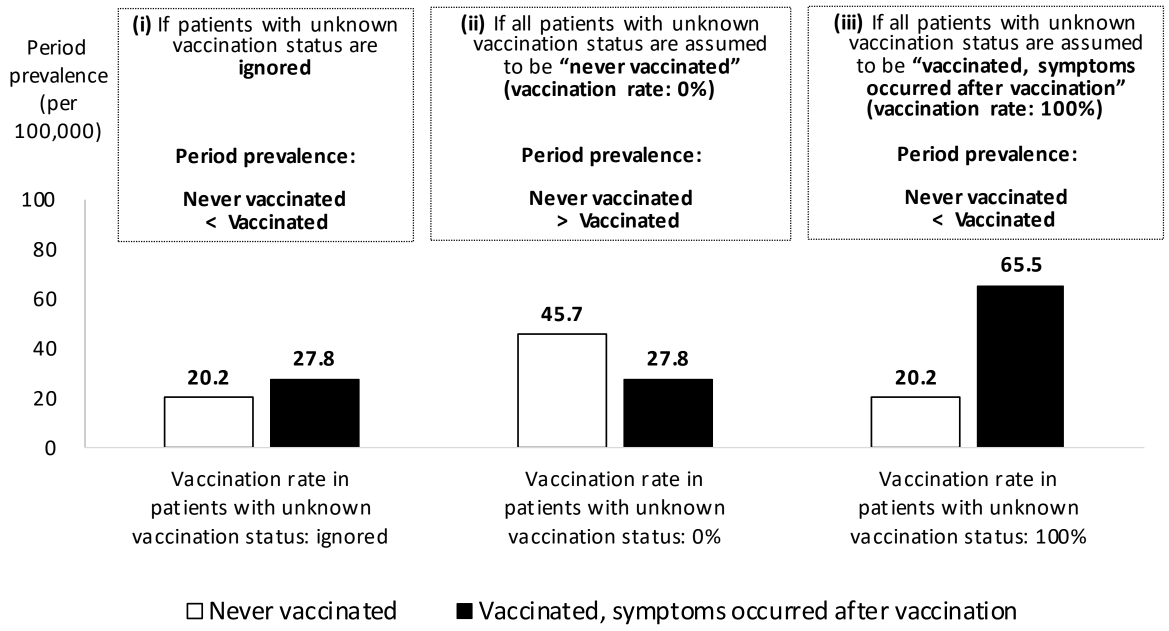

eFigure 3. Estimated period prevalence (per 100,000) during the period of July to December 2015 among girls with diverse symptoms in Group A (never vaccinated) and Group C (vaccinated, symptom occurred after vaccination): (i) shows reproduced figures from Table 5, ignoring patients in Group E (unknown vaccination status); (ii) and (iii) show hypothesized situations in which all patients in Group E were (ii) "never vaccinated" (i.e., vaccine coverage: 0%) and (iii) "vaccinated, symptom occurred after vaccination" (i.e., vaccine coverage: 100%).

eMaterials 1. Survey explanation form

Nationwide epidemiological survey of adolescent patients with diverse symptoms including pain and motor dysfunction

Did any patients who satisfied the criteria listed below visit your department for medical examination in the past 6 months (July 1 to December 31, 2015) and if "yes" what were their age- and gender- specific numbers? Kindly use the enclosed postcard to respond.

➢ **Inclusion criteria for the Survey:** Must satisfy all the criteria (1) to (4) listed below

---

(1) Age: 12 to 18 (on visiting during July 1 to December 31, 2015)

(2) Had at least one of the below-listed symptoms*

- Pain or sensory dysfunction (light, sound and smell)
- Motor dysfunction
- Autonomic dysfunction
- Cognitive impairment

(3) Symptoms in (2) persisted for at least 3 months*

(4) Both (2) or (3) influenced attendance at school or work*

---

* Please see the back of this document for additional information such as typical examples and indications.

- **Please send a response irrespective of the patients' gender and history of human papillomavirus (HPV) vaccination.**

- **Kindly gather data from all the physicians of your department based on their experience in the past 6 months, availing the opportunity offered by meetings.**
  (Kindly cooperate considering the importance of this study).

- The response form of this survey may be filled on the basis of facts recalled by the physician.
  (It is not always necessary to verify from the medical records).

eMaterials 1. Survey explanation form (continued)

Inclusion criteria for the Survey – Additional information

(2) Practical examples of the symptoms

- Pain or sensory dysfunctions (light, sound and smell)

  Pain: Joint pain, muscle pain, lower back pain, headache, abdominal pain, pain in the whole body, etc.

  Numbness: In limbs, face, trunk, etc.

  Others: Sensory loss, hypersensitivity to light, sound or smell (Examples: Spending long periods of time wearing sunglasses or headphones and heightened sensitivity to the smell of a shampoo), etc.

- Motor dysfunctions

  Dysstasia, disturbed gait, tremors, decreased grip strength, paralysis, weakness, cramps, etc.

- Autonomic dysfunctions

  Vertigo, fatigue, fainting, cold sweat, sweaty palms, low-grade fever, dizziness, ringing in the ears, nausea, vomiting, diarrhea, day-night reversal, abnormal body temperature, increase or decrease of appetite (and body weight increase or decrease in not matching with the changed appetite), etc.

- Cognitive impairments

  Reduced memory, reduced ability to learn, lowered concentration, etc.

  (Examples: Reduced proficiency in mental arithmetic, inability to write Chinese characters, reduced reading speed, and inability to recall the face of a classmate even after hearing the name).

(3) Persistence of symptoms

- If two or more symptoms have appeared off and on, judge whether they "as a whole" persisted for at least 3 months.

  (Example: The patient had pain for about 2 months followed by movement disorders without pain for about 1 month ⇒ Conclude that the "symptoms persisted as a whole for at least 3 months").

(4) Indications that the symptoms are "influencing" attendance at school or work

- Absence from school or work continuously for a week or longer
- Obvious signs of reduced capacity for activity at school or work

  (Examples: Non-participation in physical education classes, frequently spending time in the sick room, clear decline in academic performance, and inability to do work that the subject was doing earlier).

eMaterials 2. Decision process whether nor not the reported symptoms correspond to "diverse symptoms after human papillomavirus (HPV) vaccination" (detailed explanation of Table 2)

- If the doctors in charge answered that they were not able to adequately explain the patient's symptoms by the diagnoses (up to 10) that were identified/recognized by the department, the symptoms of the reported patients were classified as "corresponding to diverse symptoms". This decision was made in accordance with a claim in Japan that "there have been no diverse symptoms before the introduction of human papillomavirus (HPV) vaccine (i.e., the diverse symptoms after HPV vaccination is entirely new disease entity)".

- If the doctors in charge answered that they were able to adequately explain the patient's symptoms by the diagnoses (up to 10) that were identified/recognized by the department, and if a diagnosis that was designated as "the most explicable" by the doctor in charge included terms such as "due to HPV vaccine" or "after HPV vaccination", the symptoms of the reported patients were classified as "corresponding to diverse symptoms".

- If the doctors in charge answered that they were able to adequately explain the patient's symptoms by the diagnoses (up to 10) that were identified/recognized by the department, and a diagnosis that was designated as "the most explicable" by the doctor in charge did not include terms such as "due to HPV vaccine" or "after HPV vaccination", the symptoms of the reported patients were classified as "*not* corresponding to diverse symptoms" when the diagnosis could be clearly distinguished from diverse symptoms after HPV vaccination. Otherwise, the symptoms of the reported patients were classified as "corresponding to diverse symptoms". This decision was made in order to harmonize with the opinion of the National Expert Committee on Vaccine Safety by the MHLW that the diverse symptoms after HPV vaccine possibly attributed to functional somatic disorder. Regarding the decision on whether or not the diseases can be clearly distinguished from diverse symptoms after HPV vaccination, eight clinicians in the study group (YK, MS, YU, KH, AO, SM, SuK, SaK) independently reviewed the list of diagnoses that were designated as "the most explicable" by the doctor in charge for reported patients in the second-stage survey (a total of 201 diagnoses). A diagnosis was judged as being clearly distinguished from diverse symptoms after HPV vaccination if all eight clinicians judged so. During the reviews, no other information from the patients was provided.

- If the doctors in charge answered that they did not have any idea whether they were able to adequately explain the patient's symptoms by the diagnoses (up to 10) that were identified/recognized by the department, the symptoms of the reported patients were classified as "unknown".
